# Fine root decomposition in forest ecosystems: an ecological perspective

**DOI:** 10.3389/fpls.2023.1277510

**Published:** 2023-10-27

**Authors:** Sudipta Saha, Lei Huang, Muneer Ahmed Khoso, Haibo Wu, Donghui Han, Xiao Ma, Tika Ram Poudel, Bei Li, Meiru Zhu, Qiurui Lan, Nazmus Sakib, Ruxiao Wei, Md. Zahirul Islam, Peng Zhang, Hailong Shen

**Affiliations:** ^1^ College of Forestry, Northeast Forestry University, Harbin, China; ^2^ Key Laboratory of Saline-Alkali Vegetation Ecology Restoration, Ministry of Education, Department of Life Science, Northeast Forestry University, Harbin, China; ^3^ Key Laboratory of Sustainable Forest Ecosystem Management, Ministry of Education, Northeast Forestry University, Harbin, China; ^4^ Feline Research Center of National Forestry and Grassland Administration, College of Wildlife and Protected Area, Northeast Forestry University, Harbin, China; ^5^ State Forestry and Grassland Administration Engineering Technology Research Center of Korean Pine, Harbin, China

**Keywords:** climatic factors, decomposition, forest ecosystem, fine root, microorganism, physio-biochemical activity

## Abstract

Fine root decomposition is a physio-biochemical activity that is critical to the global carbon cycle (C) in forest ecosystems. It is crucial to investigate the mechanisms and factors that control fine root decomposition in forest ecosystems to understand their system-level carbon balance. This process can be influenced by several abiotic (e.g., mean annual temperature, mean annual precipitation, site elevation, stand age, salinity, soil pH) and biotic (e.g., microorganism, substrate quality) variables. Comparing decomposition rates within sites reveals positive impacts of nitrogen and phosphorus concentrations and negative effects of lignin concentration. Nevertheless, estimating the actual fine root breakdown is difficult due to inadequate methods, anthropogenic activities, and the impact of climate change. Herein, we propose that how fine root substrate and soil physiochemical characteristics interact with soil microorganisms to influence fine root decomposition. This review summarized the elements that influence this process, as well as the research methods used to investigate it. There is also need to study the influence of annual and seasonal changes affecting fine root decomposition. This cumulative evidence will provide information on temporal and spatial dynamics of forest ecosystems, and will determine how logging and reforestation affect fine root decomposition.

## 1 Introduction

While plant litter decomposition in terrestrial systems is one of the largest annual fluxes in global carbon (C) and nutrient cycling, the role of fine root traits relative to above-ground litter is inadequately understood (See et al., 2019). Every year, a massive amount of organic matter enters the decomposition cycle, mostly in the form of dead plant matter. When a plant loses its leaves, branches, or small roots, organic debris builds up on the forest floor, where these materials becomes a vital resource for soil organisms (Findlay, 2021). Because of foliar litters abundance and its relatively high nutrient content, it has received disproportionate attention in forest litter decomposition studies (Zhou et al., 2020).

In contrast, while researchers initially paid little attention to fine roots (which make most underground litter), research is now clarifying the important of these roots to terrestrial ecosystems (Cheng et al., 2022). Fine roots are essential to the below-ground forest biomass and contribute substantially to the soils organic matter (Hayashi et al., 2023). Understanding fine root characteristics is necessary to describe forest ecosystem C and nitrogen (N) cycles (Zhu et al., 2021). Responses by a terrestrial ecosystem to changes in its surrounding environment can be seen in the decay dynamics of fine roots, which also carry water and nutrients and perform tasks below the ground level (Cheng et al., 2022). However, in fine root decomposition research, their chemical makeup, turnover rates, and interactions with decomposer species have not been fully examined.

Fine root turnover contributes from 14-27% of net primary production (NPP) globally (Mccormack et al., 2015) and is thought to account for 33% of annual forest litter inputs and 48% of the inputs in grasslands (Freschet et al., 2017). Historically, root diameter has been used to categorize it as ‘fine’ or ‘coarse,’, with the former having a diameter of ≤2mm and the latter having a diameter >2mm (Berg and Mcclaugherty, 2020). Root litter input is highly variable across ecosystems, but in at least some it contributes the same amount of organic matter and nutrients to the soil as does foliar litter (Prescott and Grayston, 2023). Rine root death and decomposition represent a major C cost to plants, and is a potential soil C sink (Chou et al., 2022).

Fine root biomass, and its nutrient contents and rate of decomposition, can be influenced by a number of abiotic and biotic variables (Zhang et al., 2021). When organic matter is decomposed by biotic microorganisms like bacteria and fungi, more complex molecules are converted into simpler forms (Islam et al., 2022). Due to their recalcitrant character, roots with high levels of lignin (L) and secondary chemicals degrade more slowly (Phillips et al., 2023). By decomposing root tissues and promoting microbial activity in the microenvironment they create, mycorrhizal fungi and soil-dwelling biota also have an impact on decomposition (Wu et al., 2023). Microbial activity, enzyme function, and decomposition rates are affected by abiotic factors such as temperature, moisture, oxygen, N, phosphorus (P), and soil texture (Lull et al., 2023). While nutrient-rich soils promote faster decomposition due to higher biomass, well-aerated soils promote effective decomposition (Abdul Rahman et al., 2021). Understanding these factors is essential for forecasting how land use and environmental changes will affect C and nutrients cycles in terrestrial ecosystems.

In forests, tree species and functional groups differ greatly in their root characteristics (Herzog et al., 2019). The fine root biomass of boreal forests has an inverse relation with soil fertility, but has a positive association with mean annual temperature (MAT) and mean annual precipitation (MAP), and stand age (Peng and Chen, 2021). The fine root biomass of temperate forests increases with site elevation, and with higher MAT (Gao et al., 2021). Climate and soil nutrients are also linked to nutrient concentrations and fine roots contents in forest ecosystems (Pandey et al., 2023). Sometimes, the nutrient amounts released by fine roots decays exceeds that released by the decomposition of leaf residue, and a significant portion of the net primary productivity is allocated to fine roots (Yuan and Chen, 2010).

Mycorrhizal associations, which improve tree nutrition, stress tolerance, and disease protection, also occur on the network formed by fine roots (Tedersoo et al., 2020). Recent research on the function and nutrition of saprophytic fungi has led to several breakthroughs. However, how and why saprophytic fungi help break down fine roots has been infrequently addressed (Gray and Kernaghan, 2020). Recent research has revealed that, in addition to substrate quality, soil microorganisms have a significant impact on fine root decomposition (Fu et al., 2021). Additionally, the nutrient quality of litter substrate affects the growth status of saprophytic fungi, which in turn affects their abundance and diversity (Dai et al., 2021).

Moreover, the most important factor in determining how fine roots decompose depends on the root’s initial chemical characteristics (Jing et al., 2019). Thinner fine roots (0.5-1.0 mm diameter) decompose more quickly than thicker fine roots (1.0 mm diameter), possibly because the former have higher N concentration and lower C to N (C/N) ratio, which are favorable for microorganism decomposition and utilization (Lin et al., 2011). The rate of fine root breakdown may also depend on the concentrations of structural carbohydrates and non-structural carbohydrates (Zhai et al., 2023). Small molecules like glucose and starch are easy for microorganisms to break down, while macromolecules like L and cellulose can only be broken down by certain types of bacteria (Hemati et al., 2022).

For instance, microorganisms like white rot fungi (WRF) and brown rot fungi (BRF) produce ligninolytic enzymes that break down L (Theradimani and Ramamoorthy, 2022). Additionally, by altering plant histochemistry, the effect of soil nutrient availability on decomposition is more likely to indirectly (rather than directly) affect root decomposition rate (Jiang and Liang, 2022). Fertilizers applied only for a short periods can boost root detritus decomposition (thereby encouraging N release in soils for plant uptake) and contribute to long-term soil C accumulation through either additional C inputs from manures or N-induced effects on microbial activity (Fornara et al., 2020).

Furthermore, fine roots turnovers is a significant route for the transport of C and nutrients from plants to soils (Wang et al., 2020a). However, current difficulties with measuring fine root decomposition rate prevents us from precisely quantifying the scale of this activity (Sun et al., 2021). The most common approaches to assessing fine root decomposition rates use litterbags or intact cores, through their methodological efficacies have not been critically assessed (Li et al., 2022a). Litterbags and soil cores may affect the rate of decomposition and microbial activity because they separate litter from its ecosystem and may thus give misleading results about decomposition dynamics (Dornbush et al., 2002; Wu et al., 2022). Accurate total fine root decomposition measurements provide insight into subsurface C cycling processes and reduces uncertainty in soil C flux estimates. Specifically in global budgets and climate change mitigation efforts, fine root decomposition processes offer a comprehensive perspective of below-ground C dynamics, boosting soil C flow projections and enhancing C cycle management in terrestrial ecosystems (Huang et al., 2022).

Fine roots, which make up the vast majority of underground litter, are essential to terrestrial ecosystems due to their roles in redistributing water and nutrients, performing important below-ground functions, and making considerable contributions to forest ecosystem C and N cycles. Understanding fine root properties is crucial to describing forest environment C and N cycles. The biomass, nutritional content, and decomposition rate of fine roots are influenced by a variety of abiotic and biotic variables. Forecasting how changes in land use and the environment will affect cycling of C and nutrients in terrestrial ecosystems requires understanding these factors. The availability of soil nutrients can indirectly influence decomposition rates, and sparingly supplied fertilizers may hasten root debris decomposition and increase soil C levels. Accurate measurements of total fine root decomposition reduce uncertainty in soil C flux estimations and shed light on subsurface C cycling processes. Herein, we highlight the fine root decomposition process and the elements influencing that process reference for below-ground C cycle research.

## 2 Fine roots

**Figure 1:**
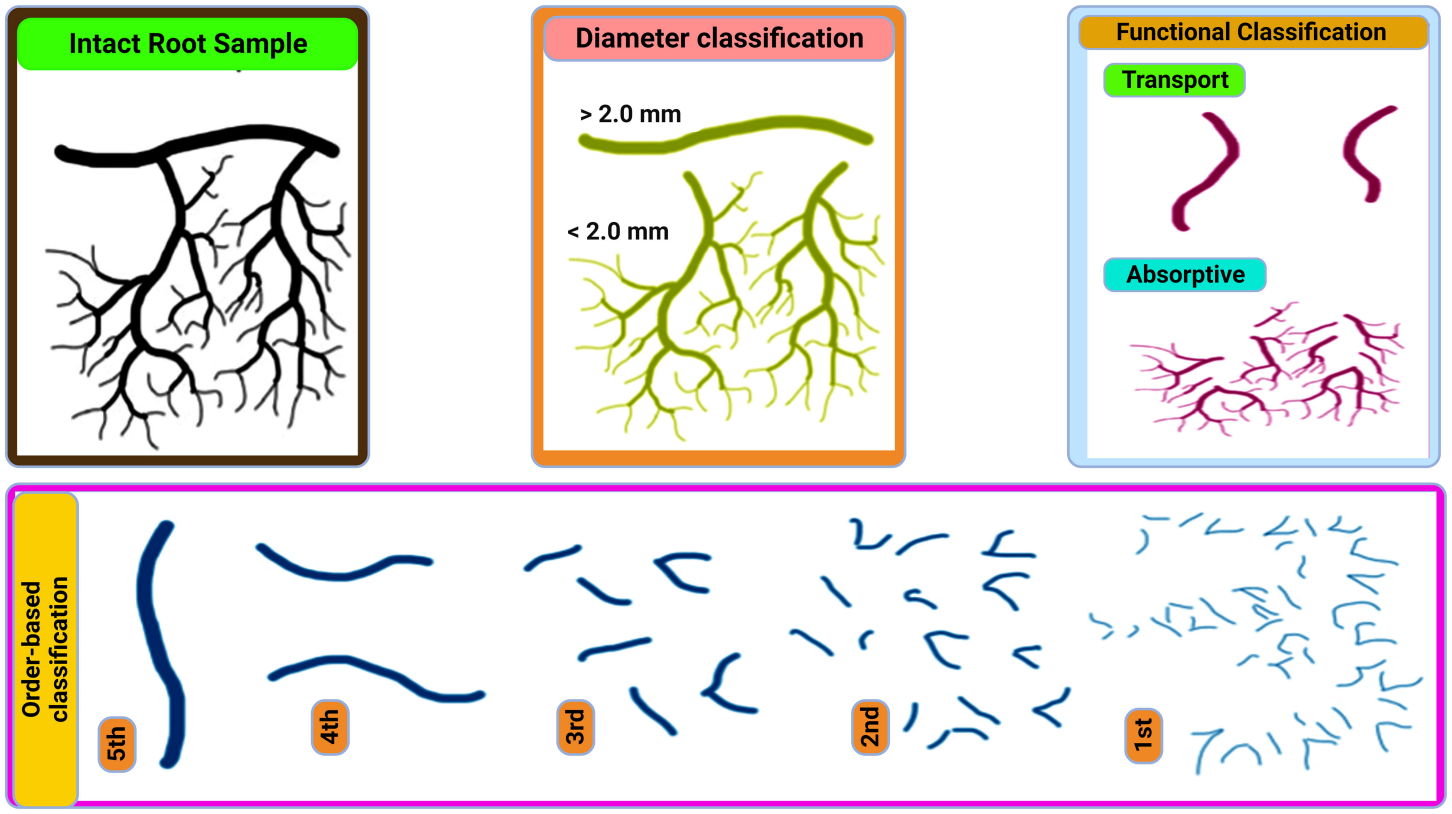
Overview of fine-root classification according to McCormack et al. (2015).

Fine roots (≤2 mm diameter) are the plants major water and nutrient uptake channels (Finér et al., 2019). They actively interface with the environment play key roles in terrestrial ecosystem processes, and make up 33% of the world’s annual NPP (Li et al., 2019). Fine roots components can be divided into long-lived transport fine roots (TFRs) and short-lived absorptive fine roots (AFRs) (**Figure 1**) (Huang et al., 2023). TFRs (root orders 4 and 5) help with long-distance transport and hydraulic conductivity by moving water and nutrients from the soil to above-ground plant components. The surface area for nutrient absorption and nutrient uptake efficiency are increased by AFRs (root orders 1-3), which are finely branched and coated in root hairs (Mccormack et al., 2015; Kou et al., 2018). Plant growth and survival are guaranteed by this division of labor, which enables plants to efficiently acquire nutrients and water while preserving structural integrity (Kou et al., 2018).

Fine root activity has the potential to alter soil physical, chemical, and biological characteristics, which can impact both individual plants and entire ecosystems (Mccormack et al., 2015). For example, as fine roots grow and proliferate, they create macropores and pathways that improve the soil’s structure and ability to hold air. These changes facilitate soil microbial mobility, enhance water infiltration, and reduce soil compaction (Wendel et al., 2022). Exudation of organic substances by fine roots (e.g., sugars, organic acids) also affects soil pH, availability of nutrients, and microbial activity. These modifications can improve N cycle processes and provide a more favorable environment for helpful soil bacteria (Keiluweit et al., 2015).

In addition, fine roots are essential to the soil C sequestration process and play crucial roles in nutrient cycling (Pandey et al., 2023). Because of the high turnover rate, the quantity of C and nutrients that are returned to the soil through fine roots is comparable to or even greater than, that which is returned through litter (Hu et al., 2020). Fine roots create a matrix that captures and stabilizes organic matter, lowering its susceptibility to quick decomposition, and thus offering physical protection for soil organic C (Waring et al., 2020).

Forest succession is likely to influence fine root decomposition, which is an important process for nutrient intake and C exchange in terrestrial ecosystems (Fu et al., 2021). Lower L contents and nutrient concentrations in early forest successional phases leads to increased decomposition rates (Morffi-Mestre et al., 2023). Slower decomposition rates are caused by changes in the fine root characteristics of old forests. Rapid decomposition releases nutrients for ecosystem productivity, but also alters nutrient cycle dynamics. Slower decomposition in mature forests helps retain nutrients, fostering conservation and long-term stability (Bhattarai et al., 2022). The world’s fine root P pool is 4.4×10^7^ Mg and its fine root N pool is one-seventh of the total terrestrial vegetation (Jackson et al., 1997). Thus, as a significant source of root bioenergy, small, quickly decomposing plant fine roots (<2 mm diameter) are crucial to forest nutrients cycling (Rodtassana and Tanner, 2018). Understanding these processes is crucial for sustaining and restoring forest health, while controlling nutrient cycling in shifting landscapes, through ecosystem management (Rai, 2022).

## 3 Fine root decomposition

Fine-root decomposition is a material exchange process with the environment that involves soil biological metabolism and the absorption and release of chemical elements as a result of soil leaching and breakdown (He et al., 2019). Fine root decomposition regulates nutrient release, carbon dioxide (CO_2_) emission, and soil organic matter (SOM) synthesis in forest ecosystems (Jacobs et al., 2018; Babur and Dindarolu, 2020). In the initial phase of decomposition, environmental factors, such as soil temperature, soil moisture content, and substrate breakdown cause rapid eluviation of carbohydrates and other soluble compounds (He et al., 2019).

Later, biological action dominates the decomposition process, breaking down the soluble components and leaving insoluble molecules (such as L and cellulose) for sluggish microbiological degradation (He et al., 2019). Both bacteria and fungi, which carry out distinct tasks within their respective groups, are microorganisms engaged in the breakdown process (Zhao et al., 2021a). As early decomposers, bacteria use enzymatic activities to reduce complex organic matter to simpler molecules. In the initial decomposition stages, they serve a crucial role in making organic matter accessible to other organisms. Later decomposition phases are aided by fungi, which are effective at dissolving complex substances like L and cellulose (Bonfante and Genre, 2010). Decomposition dynamics are further influenced by mycorrhizal fungi, which create symbiotic connections with plants and aid nutrient intake (Schädler et al., 2010).

Fine root decomposition may differ at the species level depending on traits related to aspects of the plant economics spectrum like growth form (e.g., woody vs. herbaceous, broadleaf vs. conifer), nutrient acquisition strategy (i.e. mycorrhizal association), leaf lifespan of woody plants (i.e. deciduous vs. evergreen), and herbaceous life cycle (i.e. annual vs. perennial) (See et al., 2019). The main factors affecting fine root decomposition include substrate quality and soil environmental parameters, including soil temperature, humidity, pH, bulk density, and soil microorganisms (Zhang et al., 2019a; Zhang et al., 2019b; Tanikawa et al., 2023). The dynamic structure and function of the microbial community will adapt to the degradation process (Yan et al., 2022). Indeed, through underground root decomposition and nutrient humidification, soil microbial communities enzyme systems contributes to about 90% of forest ecosystem biological cycle (Finzi et al., 2006).

However, significant variation remains unexplained, both worldwide and regionally, as plant tissue breakdown rates are positively associated with MAT and MAP (Santos and Herndon, 2023). Higher precipitation provides moisture, while warmer temperatures increases microbial activity (Wahid et al., 2020). These correlations might differ among environments due to variables like soil quality and litter quality (Hermans et al., 2020). Understanding these relations emphasizes the significance of considering MAT and MAP as significant drivers in C and nutrient cycling, and may aids forecasting fine root decomposition’s responses to climate and environmental changes.

**Figure 2:**
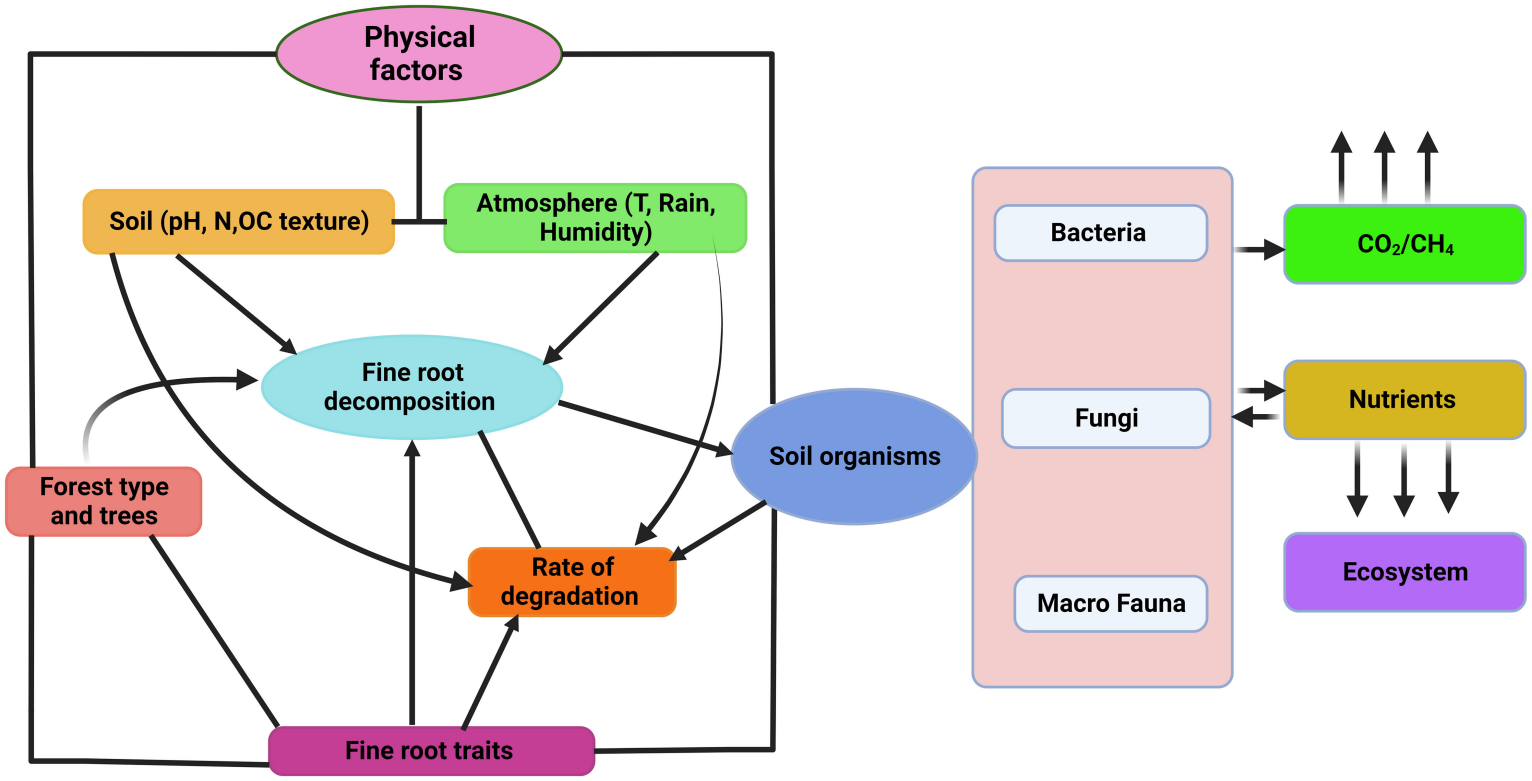
Diagrammatic representation of factors affecting fine root decomposition in forest ecosystem.

In temperate and boreal woodlands, changes in fine root biomass have a considerable impact on N cycling and ecosystem function (Cornejo et al., 2023). Fine root biomass is inversely associated with soil fertility in boreal forests, but positively correlated with MAT, MAP, and stand age (Yuan and Chen, 2010). Fine root biomass increased with site elevation in temperate forests, but declines with MAT (Esser et al., 2012). Fine root biomass changes impact both the ability of boreal forests to store C, and nutrient cycling and availability in temperate woodlands (Meena et al., 2023). Higher fine root biomass can make it easier for plants to get the nutrients they need, which can help them grow and produce more (Gao et al., 2021). However, too much biomass can cause nutrients to become immobile, which can slow plant growth and production (Li et al., 2021). To maintain ecosystem function and nutrient cycling in these forest types, it is essential to understand and manage fine root biomass changes. Fine root degradation processes are shown in **Figure 2**.

## 4 Factors affecting fine root decomposition

Terrestrial environments nutrient cycles rely heavily on fine root decomposition (Zhao et al., 2023b). The two primary variables affecting decomposition are climate (**Table 1**) and fine root chemical properties (Guo et al., 2021). Root decomposition rate is positively associated with both global MAT and MAP (See et al., 2019). How roots’ chemical properties affect the decomposition rate also depends, in turn, on root factors. Increases in root N and decreases in root L concentrations both enhance the fine root decomposition rate (Bonanomi et al., 2021). Mycorrhizal symbioses can be formed between more than 90% of all woody plants (Chen et al., 2016). Root morphological features and chemical composition may influence the pace of fine root degradation in trees that have either arbuscular mycorrhizal (AM) or ectomycorrhizal (ECM) relations (Chen et al., 2018).

**Table 1:** A comparative table on root decomposition in different climatic zones.

Climatic Zone	Average Temperature (°C)	Moisture Levels	Decomposition Rate	Main Factors	Reference
Tropical	25-30	High	Rapid	Warm temperature, Enough rainfall, Abundant litter input, Microbial diversity, and mycorrhizal association	(Powers et al., 2009; Guerrero-Ramírez et al., 2016; Zhao et al., 2023b)
Temperate	5-20	Moderate to High	Moderate	Seasonal climatic conditions, Coniferous litter, mycorrhizal fungi, Soil invertebrates	(Solly et al., 2014; Zhao et al., 2023a; Zhao et al., 2023b)
Mediterranean	15-25	Moderate to low	Moderate to slow	Dry summer, Mild winter, Root chemistry, and Root order, Specific microbial community	(Zhang and Wang, 2015; Sun et al., 2016; Bonanomi et al., 2021)

### 4.1 Climatic factors

Given that temperature and precipitation are key influences of plant growth and decomposer activity, root decomposition is likely to be highly responsive to variations in these two parameters (Bonato et al., 2023; Semeraro et al., 2023). In addition, the availability of nutrients and C sources influences the response of microorganisms to climate change (Sullivan et al., 2019). It is important to consider the general reactions of C stocks in terrestrial ecosystems to changes in climatic condition changes, especially temperature and precipitation, as well as the consequences of synergies of these factors (Fanin et al., 2022b). If the nutrients and effective C sources required for microbial activity are lacking, microorganisms will not sensitive to temperature changes, and their effect on decomposition will be weak (Baldrian et al., 2023). Soil temperature and humidity can alter microbial activities such as fungal hyphae expansion, fruiting body formation, spore germination, and release, and subsurface ecological processes such as root decomposition (Kvasko et al., 2022). However, annual air temperature is also linked to the root decomposition rate globally (Guo et al., 2021). At all sites assessed, the rate of C mineralization decreases with soil depth and time, and increases with temperature (Jia et al., 2022). Fine roots vitality’s is reduced by 90% for every 10°C increase in annual average temperature (Eissenstat and Yanai, 2002). The primary causes of this phenomenon are: (1) As temperature rises, root respiration increases (Wang et al., 2021a); (2) Soil N mineralization speeds up (Lakshmi et al., 2020); and (3) Bacterial activity is improved in warm soil (Nottingham et al., 2019).

A primary causes of fine roots concentrations in the surface layer is thought to be the quick decrease in soil temperature of descending layers; the higher surface layer temperature also encourages decomposition and increases soil nutrients, favoring fine root growth (Sihui et al., 2022). Higher soil temperatures not only increases fine root biomass, it leads to their distribution in deeper soil (Jarvi and Burton, 2020). With decreased or increased precipitation, fine root biomass, production, and decomposition increase, decrease, or remain unaltered across plant types and soil depths (Wang et al., 2020b). A plant’s capacity for water absorption and transmission is influenced by its fine root diameter and length. Reduced precipitation slows root growth due to nutrient deficits (Yan et al., 2019).

According to resource economics theory, fine roots may display a strategy that increases resource acquisition in areas with ample precipitation by lengthening their total root length, specific root length, and specific root area (Weemstra et al., 2017). Reduced precipitation greatly boosts fine root decomposition but has no impact on root production (Zhang et al., 2018b). Additionally, pulse precipitation mechanics encourage microbes to rapidly decompose SOM over short periods, causing significant CO_2_ release into the atmosphere (Xu et al., 2020).

After precipitation, the rate of microbial mineralization might rise by a factor of several to 10, leading to increased ecosystem nutrient availability and enhanced microbial activity (Sponseller, 2007). Soil microbial activity peaks as rainwater enters the ground, and because it remains elevated for a longer time, more CO_2_ is produced. After reaching peak activity levels, soil bacteria quickly degrade abundant organic substrates, releasing CO_2_ in much greater quantities compared with that of tropical forests (Zhaoxia et al., 2021). Increase CO_2_ emissions brought on by rainfall is responsible for about 20% of total annual soil CO_2_ emissions (Austin et al., 2004). This occurs rainwater introducing new organic matter (e.g., plant remains, litter) to soil, effectively promoting microorganisms activity’s (Javed et al., 2022). This increased microbe metabolic activity following entry of fresh organic material increases, the rate at which organic matter decomposes; thus increased microbial activity causes emission of more CO_2_ from the soil into the atmosphere (Mehmood et al., 2020). The precipitation-induced priming effect, which also emphasizes how transient environmental events like rainfall can significantly impact annual C emissions from terrestrial settings, demonstrates the dynamic, interrelated nature of ecosystem C cycles.

### 4.2 Substrate quality

Root substrate quality is among the most important factors of root nutrient cycling (Jing et al., 2019). Different root diameter sizes exhibit notable variations in morphology, physical-chemical characteristics, and stoichiometric ratios as a result of the root branching hierarchy (Han et al., 2019). The rate of element release or immobilization during the root decomposition process may therefore be mediated by diameter-associated variations in root substrate chemistry (Wang et al., 2014). However, previous studies have demonstrated that initial quality primarily mediates root decomposition (See et al., 2019). While fine roots are often nonwoody and ephemeral absorptive roots, coarser roots function as conduits, storage sites, and physical anchors for nutrients. Thus, the breakdown patterns may change based on their functional differences (Guo et al., 2008). Furthermore, fine root decomposition is typically largely slowed by high L concentrations (Da et al., 2017). Early studies comparing root and leaf chemical features showed that roots have higher concentrations of L, and the lower L content in leaves is frequently cited as the main factor for their rapid decomposition (Fujimaki et al., 2008).

Other initial litter substrate qualities, such as N, P, calcium (Ca), and L/N ratios, have been identified as regulators of mass loss and nutrient cycling rates in comparisons of composition among various tree species (e.g., broadleaves vs. conifers, N-fixing vs. non-fixing) (Aponte et al., 2012; Li et al., 2014). These initial traits support the theory, expressed in multiple studies of various ecosystems, that the L: N ratio control litter decomposition alone or in combination with other factors (Berg, 2014). Leaf litter with a low initial L: N ratio produces a higher fraction of slowly dissolving organic matter in late decomposition stages, while N is negatively linked with species-specific decomposition limit values (Berg, 2014; Hobbie, 2015). Thus, higher decomposition rates can be expected from plant litter with lower C/N or L/N ratios.

### 4.3 Roles of soil properties

Roots can die and disintegrate at any time of year, continually supplying soil nutrients and playing an important role in the biogeochemical cycle (Sardar et al., 2023). Root exudation, mortality, and shedding are other important contributors to soil C pool replenishment (Liu and Liao, 2022). Root decay thus becomes a primary source of subterranean nutrients and organic materials. Root decomposition releases significant volumes of organic matter and nutrients into the soil, where they serve an essential part in reviving and boosting soil fertility, enhancing forest productivity, and ensuring continued, sustainable growth of forest ecosystems (Gulati and Kaur, 2023). Root activities significantly impact both the rate at which organic C is accumulated in the soil and overall C circulation throughout the biosphere (Kowalska et al., 2020). Through interpenetration, entanglement, and cementation, roots can effectively enhance soil structure and stabilize organic C in soils (Dijkstra et al., 2021). The initial phase of fine root decomposition is rapid due to high root quantities of soluble carbohydrates, which are easily lost by leaching and then used by soil microbes, which further speed up root decomposition (Ahmed et al., 2022). Furthermore, accumulation of L and other difficult-to-decompose compounds in fine roots during the late decomposition stage results in a lower root decomposition rate (Wambsganss et al., 2021; Li et al., 2022b). Soil pH also impacts plant enzyme activity’s, with mean soil acidity reducing soil microorganism numbers, which slows the organic matter decomposition rate, and thus prevents litter decomposition (Fu et al., 2021; Fanin et al., 2022a; Li et al., 2022b).

The root N-release technique is more complicated. The various stages of root decomposition either release N into the soil or increase its availability. After plant decomposition, soil N concentration increases to 120–150% of its original value (Zhao et al., 2021b). There is also an enrichment-release pattern of soil nutrients, with total soil N concentration lower in the summer and higher in autumn, during fine root decomposition periods (Jiang and Liang, 2022). Others have asserted that during plant litter breakdown, dynamics of N, P, and heavy metal elements (e.g., Fe, Al, Mn, Pb, Cu, and Zn) typically demonstrated enrichment-release mechanisms (Gong et al., 2020; Alon et al., 2021; Bhattarai et al., 2022). N is released during the initial root decomposition stage, slightly increasing soil nitrate nitrogen levels. However, rises in warmth and rainfall have led to more modest increase in ammonium nitrogen content. Temperature and moisture variations across seasons have major impact on organic N mineralization, nitrification, and denitrification processes, as well as accessible N quantities (Dong et al., 2020; Jiang and Liang, 2022). Application of N or P fertilizer has been shown to increase and develop decomposition rates of fine roots at longer-term treatment sites, whereas the rates of thin roots at the shortest-term sites did not respond significantly; this may be related to soil microbial activity after fertilization, in which N and P limit C sources rather than the opposite (**Table 2**) (Titus and Malcolm, 1987).

**Table 2:** Effects of nitrogen deposition on fine root decomposition in forest ecosystems.

Fine root	Research sites	Nitrogen deposition	Nitrogen application frequency (a)	Major finding	Reference
Decomposition	Northern hardwood forest stands, Michigan, USA	N deposition (n = 3); ambient N + 30 kg N ha^−1^ year^−1^ as NaNO_3_ pellets in six equal applications	20	Decomposition of fine root litter is impeded by N deposition, and SOM contributions from lignin-derived fine root chemicals are raised.	(Argiroff et al., 2019)
	Erguna River Basin, in the northeastern part of Inner Mongolia, China	Control, 10: 0, 7: 3, 5: 5, 3: 7, and 0: 10 (IN: NH_4_NO_3_, ON:CO(NH_2_)_2_, and C_2_H_5_NO_2_)	2	Decomposition rates for fine roots of all species were increased after being exposed to exogenous N in either IN or ON forms.	(Dong et al., 2020)
	In Heilongjiang Province, Northeast China, National nature reserve called Liangshui.	Control, Low-N, Medium-N, and High-N (0, 20, 40, 80 kgNha^-1^yr^-1^)	3	Root decomposition was slowed down by nitrogen deposition, which also improved soil retention of nutrients and carbon.	(Geng and Jin, 2022)
	Shaanxi Tielongwan Forest Farm Oil pine	0, 30, 60, 90 kg·hm^−2^·a^−1^ (CO(NH_2_)_2_)	3	It has a certain stage, and the rate size presents the characteristics of first fast and then slow.	(Gu and Wang, 2017)
Decomposition	Songyugou watershed in the Loess Plateau region, Shaanxi Province, China	0, 3, 6, and 9 g N m^−2^ y^−1^ (NH_4_NO_3_)	2	The decomposition of fine roots, which is influenced by N deposition, alters the biogeochemical processes of forest ecosystems.	(Jing et al., 2019)
	Jiangxi Tropical Wetland Pine Forest	0, 40, 120 kg·hm^−2^·a^−1^ (NH_4_Cl and NaNO_3_)	6	It promotes the absorption of fine root turnover rate but does not accelerate the transport of fine root turnover rate.	(Kou et al., 2018)
	Heilongjiang temperate typical forest ecological system	0, 100 kg·hm^−2^·a^−1^ (NH_4_NO_3_)	9	The quality loss of fine roots reached 30% ~ 50% on day 516, and then the change in mass residue rate was relatively flat.	(Li et al., 2017)
	Northeast Laoshan temperate forest	0, 100 (NH_4_NO_3_)	4	The initial decomposition rate of tertiary and quaternary roots was increased, but it had an inhibitory effect on primary and secondary roots.	(Sun et al., 2015)
	Northeast Daxing’an Ridge deciduous pine forest	0, 25, 50, 75 kg·hm^−2^·a^−1^ (NH_4_NO_3_)	5	The fine root turnover rate tends to decrease, possibly due to slower subsurface C cycling due to nitrogen deposition.	(Yan et al., 2017)

Moreover, Ca^2+^ plays a unique role in the decomposition process. It is not only an essential metabolic component for microorganism growth, Ca^2+^ in the root can also be used by fungi and heterotrophic bacteria to form oxalate, which provides nutrients for microorganism metabolisms in conditions that would be otherwise unfavorable (Grabovich et al., 1995). Although Ca^2+^ concentration influences how quickly root decompose, its role in how quickly fine roots decompose remain unclear.

### 4.4 Mycorrhizal association

**Figure 3:**
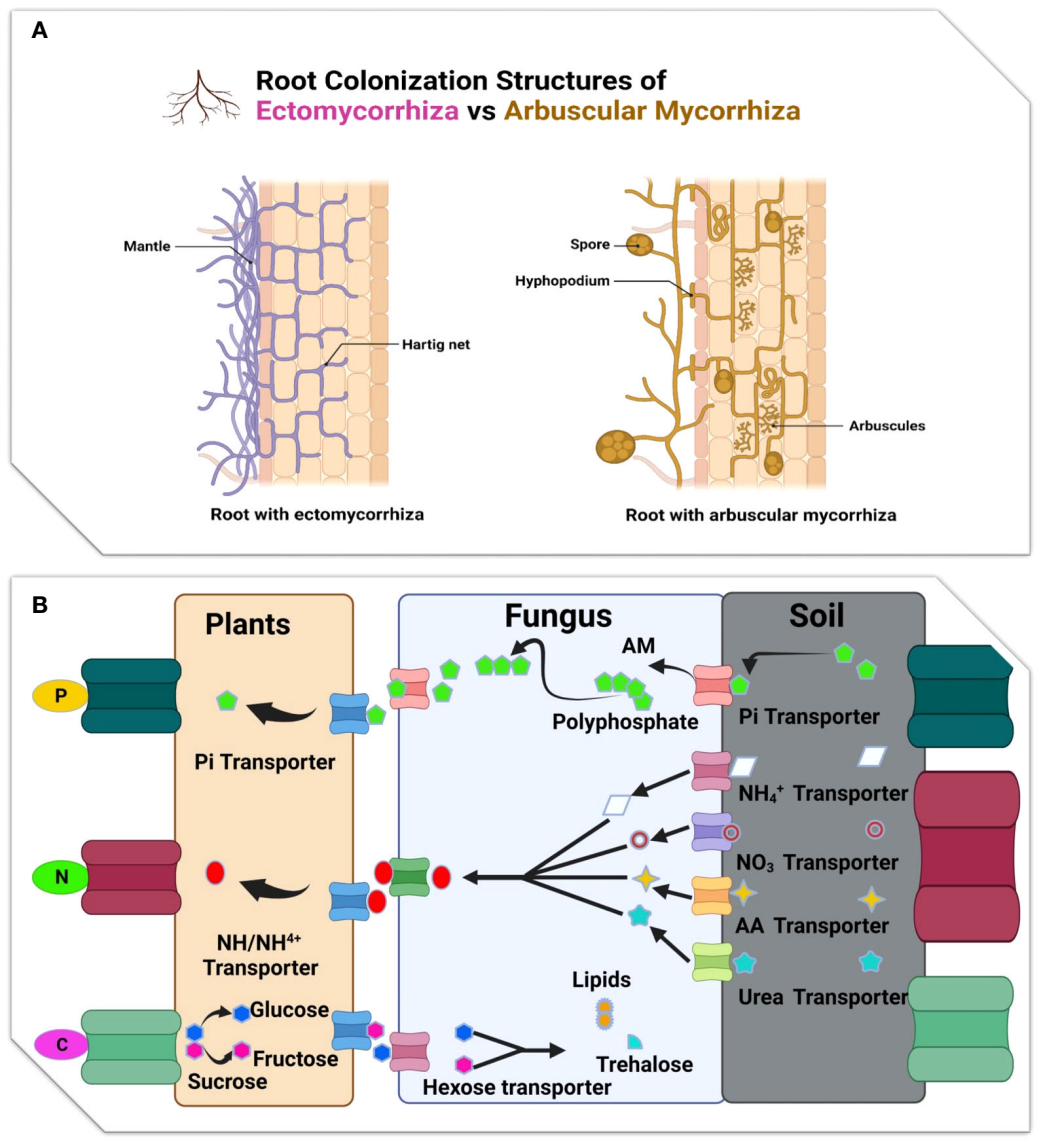
**(A)** Illustration root colonization structure of Ectomycorrhizal and Arbuscular mycorrhiza: While the Hartig net grows around epidermal cells (green), the ectomycorrhizal fungus surrounds the root tip with a thick mantle of tightly packed hyphae. The root tip is often not colonised by arbuscular mycorrhizas. A hyphopodium is produced on the root epidermis by the growth of hyphae from a spore. The formation of arbuscules, or tiny fungal trees, inside inner cortical cells, is the result of intraradical colonisation, which takes place both intracellularly and intercellularly. **(B)** Diagram showing the primary nutrient transfer pathways that take place during the EM and AM symbiosis.

Mycorrhiza plays a significant part in soil C and N cycle, and in root formation in the plant-soil system (Hawkins et al., 2023). AMF colonization is thought to improve aboveground litter decomposition but has little influence on root litter (**Figure 3**) (Schädler et al., 2010). N is regarded as a key component in the mycorrhizal influence on breakdown processes. Through the effective utilization of decomposition byproducts, AMF can accelerate the pace of litter decomposition and acquire inorganic N produced from the litter during its breakdown (Urcelay et al., 2011).

However, by altering the proportions of soil ammonium-N and nitrate-N, AMF can speed up the process of litter decomposition (Liu et al., 2021). Conifer fine roots deteriorate more slowly than those of broadleaved plants, whereas the fine roots of non-woody plants decay more slowly than those of woody plants (Phillips et al., 2023). In addition, the discovery that fine root of woody ECM and ericoid species degrade more slowly than those of AM species adds to the growing list of biogeochemical variations between these forest types (Craig et al., 2018; Zhang et al., 2018a). Several isolates of ECM fungi decompose much faster than fine root (*P. resinosa*) seedlings. Depending on the fungus isolate, ECM colonization either does not affect root decomposition or significantly sped root breakdown. Tested isolates include *Srobilomyces floccupus* (SC111), *Cladophialophora* sp. (SC052), *Lactarius oculatus* (SC076), *Amanita rubescens* (SC009), *Suillus intermedius* (SC065), *Amanita pantherina* (SC004), *Amanita citrina* (SC070), *Russula* sp. (SC079), *Amanita muscaria* var. *formosa* (SC059), *Tylopilus felleus* (SC121), and *Amanita brunnescens* (SC007 and SC040) (Koide et al., 2011).

The primary cause of root decomposition is difference in soil water and temperature differences during the initial decomposition stage. Although there are slight differences between fine root decomposition rates of *A. halodendron* at different starting times, other factors, such as variability in soil moisture and its interaction with soil temperature, are likely to have a greater impact on the root decomposition process overall (Luo et al., 2016; Luo et al., 2020). Consistent with those findings, the presence of mycelia hastens fine root decomposition (Pritsch and Garbaye, 2011). Mycelia can increase the activity of soil bacteria by providing them with fresh C, speeding up the breakdown of soil organic carbon (SOC), especially the pool of inert C (Zhang et al., 2018b). Root exudates are thought to play a significant role in litter decomposition and the soil N cycle, with their secondary metabolites inhibiting soil microbes to prevent SOC decomposition (Yin et al., 2014; Zwetsloot et al., 2018).

Additionally, plant-soil feedback processes can impact ecosystem performance because primary metabolite exudation processes are linked to plant nutrition strategies and have substantial effects on SOM decomposition by soil microorganisms (Canarini et al., 2019). Thus, a mechanistic understanding of the function that roots exudation of metabolites and plant-microbe interactions play in nutrient intake and plant community dynamics is essential for developing more efficient root decomposition dynamics in forest ecosystems.

#### 4.4.1 Saprophytic fungi in fine root decomposition

Saprophytic fungi are a crucial component of soil ecosystems; as the primary litter decomposers, they play a key role in nutrient cycling and plant community health (Sudharsan et al., 2023). Roots in forests are primarily decomposed by saprophytic fungi (Purahong et al., 2016; Baldrian, 2017), some of which (i.e., primarily basidiomycetes) have genes encoding enzymes (Riley et al., 2014), which can disrupt plant cell walls during the decomposition of forest fine roots and subsequently affect organic matter breakdown, C fixation, and N/P conversion.

Extracellular oxidative reductionase-laccase, a peroxidase is actively secreted by WRF, allowing it to degrade L (Kellner et al., 2014). Manganese peroxidase (MnP) is the only peroxidase that requires manganese (Mn^2+^) as a substrate; however, other peroxidases, such as versatile peroxidase (VP), are also applicable. Though Mn^2+^ is oxidized, it is not required for enzymatic activity, such as L peroxylase, and general oxidase. The function of additional peroxidases is unknown, however, MnP, V), and LiPs are L breakdown enzymes (Lundell et al., 2010; Hatakka and Hammel, 2011; Riley et al., 2014); the enzyme system responsible for breaking down cellulose in decomposition is shared by Apiforma and WRF. Soft rot fungi (SRF) don’t have LiPs, which helps break down L by releasing laccase. BRF extracellular carboxyl radicals break down the woody cell wall first, releasing small molecular weight oxidants through a Fenton-type chemical reaction called ‘random attack’, which breaks down substrates and speeds L breakdown (Leonhardt et al., 2019; Venkatesagowda, 2019).

During litter decomposition, WRF and BRF release many organic acids to make an ideal environment for L decomposition (Leonhardt et al., 2019). Copper-ion-dependent polysaccharide monooxygenase can increase the ability of glycoside hydrolases to break down cellulose by oxidative cleavage, increasing other cellulases activities (Long et al., 2022).

## 5 Degradation patterns of biomass materials in fine root

### 5.1 Cellulose and hemicellulose

In the forest subsurface, fungi play a crucial role in litter decomposition. To prevent polysaccharides from being degraded by microorganisms, deciduous cell walls are rich in cellulose and hemicellulose (Liers et al., 2010), and only basidiomycetes and a few ascomycetes fungi are capable of decomposing their cell wall structure (Stokland et al., 2012) to separate organic materials. Cellulose is a biopolymer polymer generated by β -1,4 glycosidic linkages linked to glucose that is difficult to disintegrate spontaneously (Klemm et al., 2005). To obtain the energy and nutrients required for mycelium growth and respiratory metabolism, saprophytic fungi ‘attack’ the cellulose microfibril structure by releasing endoglucanase, which breaks down particular macromolecules into small molecules and makes them soluble substances (Edwards et al., 2008).

The polymer known as hemicellulose is made up of xylan, xylogcan, galactosylmannan, and other similar substances (Edwards et al., 2008). Saprophytic fungi are most active in the early decomposition of hemicellulose; xyloglucan decomposition requires enzymes similar to cellulose cleavage activity, as well as xyloglucan-specific endoglucanases and exoglucanases (Master et al., 2008); Galactomannan is abundant in the cell wall of coniferous plants, the skeleton of which consists of D-mannose residues linked by β-1,4 bonds (Edwards et al., 2008). Fungi can also secrete endomannanase, β-mannosinoside enzymes such as enzymes achieve complete decomposition of them (Master et al., 2008).

### 5.2 Lignin

The secondary cell wall is coated with L, the second-most ubiquitous biopolymer after cellulose, which gives the wall its structural stability and hydrophobicity (Sreejaya et al., 2022). Syringyl (S), vanillyl, guaiacyl (G), and p-hydroxyphenyl are the three monomeric units making up L. Ls are rich in G-units that form branching Ls, which are more resistant than linear S-rich Ls, and the L matrix in plants is a function of the proportionate abundance of monomers (Jiang et al., 2023). Degradation of L is considered a process that varies among the three primary categories of decomposers, or fungi that cause white rot, soft rot, and brown rot (Floudas, 2021). Although organisms use a wide variety of enzyme pathways for L breakdown, only a subset has been thoroughly characterized. Only the WRF *Phanerochaete chrysosporium* currently has a well-described mechanism for L breakdown (Pandharikar et al., 2022). Several physiological phenomena, including synthesis of the ligninolytic enzyme *P. chrysosporium* system, appear to be induced by N deprivation (Reineke and Schlömann, 2023). Almost all WRF produce MnP, which may lead to the formation of an ecological niche based on Mn as a limiting nutrient (Baker et al., 2019). Although our understanding of *P. chrysosporium’s* ligninolytic system exceeds that of most other white-rots, it appears that the systems are species-specific (Hatakka, 2001). In WRF *Ganoderma lucidum* generates MnP in a medium containing poplar wood but not one containing pine wood (D’souza et al., 1999).

Additionally, BRF can drastically alter the L molecule but cannot fully mineralize substance; rather, they primarily degrade the cellulose and hemicellulose components of wood (Devi et al., 2023). Residual L after cellulose decomposition by BRF can resist further breakdown, forming humus, and has been associated with soil C pools, and play an important role in terrestrial C sequestration (Bonner et al., 2019). WRF and BRF are thought to have similar break-down methods. In both cases, it is important for hydroxyl radicals to form and attack wood parts, a process aided by high-oxygen tensions (Hatakka, 2001). Radicals generated by BRF can remove methoxyl groups from L and produce methanol, leaving primarily modified L as residues (Venkatesagowda and Dekker, 2021). Brown-rotted Ls are structurally different from the native version in that they have more phenolic hydroxyl groups and fewer methoxyl groups (Wei et al., 2023). Instead of breaking down L, SRF seek to soften wood by dissolving the cell walls central lamella. Ascomycetes and deuteromycetes, which make up the majority of SRF, flourish in wood with high moisture content (Philippe et al., 2022).

## 6 Analytical methods to study fine root decomposition

Our current understanding of elemental fluxes through decaying fine roots has been gleaned from the litterbag technique, which is by far the most widely used method for monitoring fine-root decay (Silver and Miya, 2001). However, whether fine roots are appropriate for litterbags use has not been confirmed (Wu et al., 2022). Fine-root decay rates recorded using litterbags appear to be too low to account for fine-root turnover rates measured with minirhizotrons and other *in situ* methods (Wu et al., 2022). The process of preparing litterbags often includes removing roots from soil and rhizosphere communities, washing and drying them, and frequently incorporating living root material (Li et al., 2022a). It follows that the rates of mass loss and nutrient turnover estimated from litterbag data may be inaccurate.

Additionally, in several significant aspects, the intact-core technique differs from litterbag research. To begin, the initial mass of roots that are included within each core is unknown. This is due to the fact that cores are taken from field soils and are preserved as whole units. Consequently mass loss estimates from undamaged cores are derived from shifts in population means over time, rather than from variations in individual samples, as is possible with litterbags (Li et al., 2022a). Although fine roots naturally senesce, the intact cores that contain both living and dead roots represent the relative decomposition rates of freshly removed live and dead roots (Li et al., 2022a).

Two novel ‘balanced hybrid’ modeling methods may provide better approach to determining how much fine root decomposition takes place in forests. In this approach, minirhizotrons and sequential soil coring procedures are used to determine how fine root dynamics are affected by the absence of any soil modification. Insights into the dynamic nature of fine root systems were gained through the use of minirhizotrons to estimate fine root turnover and mortality rates. To obtain a more complete picture of the distribution and makeup of fine roots, sequential soil coring was also used to measure the standing biomass and necromass of those roots. A mass balance model was used to calculate the overall amount of fine root decomposition, accounting for important factors like the fine root turnover rate, mortality rate, observed fine root biomass, and necromass. This method enabled a more complete assessment of belowground C cycling and nutrient dynamics in the studied ecosystem by integrating data from minirhizotrons and soil coring, providing a more holistic understanding of fine root dynamics such as growth, mortality, and decomposition (Kou et al., 2018; Li et al., 2020).

## 7 Future research perspectives

For hundreds of years, partial above ground litter has been the research subject. In contrast, fine roots researches has a recent history, and early studies tended to concentrate on fine root yield and growth distribution rather than their decomposition rate. To fully appreciate the importance of forest ecosystems, we must understand the function of fine roots in nutrient cycling, SOM dynamics, and C sequestration (Wang et al., 2021b). For synthesis, we must also collect extensive data on the variables that control fine root breakdown at different scales. While many studies have examined the role of biotic and abiotic factors in fine root decomposition (Zhang et al., 2021), comparing their results is challenging due to problems with inconsistent research methods (Solly, 2015), like use of net bags with varying pore sizes (**Table 3**). Few studies have addressed fine root decomposition around the world (Freschet et al., 2021), though not nearly enough to construct regional or global models and even less is known about how root decomposition reacts to global change and anthropogenic activities like forest conversion and forestry management.

**Table 3:** Advantages and Limitation of different root litter decomposition methods.

Methods	Advantages	Limitation	Reference
Litterbags	It is simple and inexpensive to use, can determine the decomposition rate of specific species, and may be used for all forest types.	Decomposer community composition, substrate unrepresentation, and the living root effect are affected by the experimental duration, length, and sampling regime.	(Bonanomi et al., 2021; Li et al., 2022a)
Intact core	Capable of preserving the rhizosphere’s integrity.	Limited to monodominant plantation forests, unrepresentative substrates, altered decomposer community composition, no living roots, low temporal precision, and labor-intensive.	(Freschet et al., 2021; Li et al., 2022a)
Mass balance model	Fine root decomposition can be estimated in detail with the help of the mass balance model, which takes into account variables such as turnover, mortality, biomass, and necromass. It compares ecosystems and treatment conditions using long-term breakdown trends and quantitative estimations. It provides a more detailed view of the process by factoring in microbial activity, ambient variables, and root quality. This adaptable model may be used in a wide range of environments to better understand ecological processes.	A mass balance model is intricate and data-intensive, needing precise measurements of elements like biomass and root turnover. In dynamic ecosystems, it may be misleading to rely on assumptions like constant turnover rates and steady-state conditions. For successful modeling, especially in remote or less-researched locations, precise parameter data is essential.	(Li and Lange, 2015; Li et al., 2020)

There are differences in decomposition patterns and factors controlling fine roots buried underground, despite the chemical composition of fine roots being identical to that of aboveground litter (Wang et al., 2021c). This fact makes improves our ability to study novel research methods and research directions of aboveground litter decomposition. We submit that future studies should priorities the following areas: 1) Investigating fine root decomposition in various ecosystem types to reveal their dynamic changes and the factors influencing their decomposition (Bonanomi et al., 2021); 2) Root decomposition pattern under anthropogenic disturbances and global climate changes and their effects on C sequestration (Panchal et al., 2022); 3) Function of soil organisms in fine root decomposition, as revealed by isotope tracking of fine root decomposition products and the fine root portions consumed by soil microorganisms (Prescott and Vesterdal, 2021); 4) Innovative techniques, such as solid-state 13C nuclear magnetic resonance spectroscopy (13C-NMR spectroscopy), to study fine root decomposition at the molecular level (Chu, 2020); 5) Model development to characterize decomposition processes and forecast decomposition rates based on suitable data sets, and 6) Assess how the diverse community structure of saprophytic fungi breaks down organic matter and recycles nutrients in the forest ecosystem (Zhao et al., 2020). To these ends, the General Unified Nametagging for Fungi (GUNGuild), GeoChip, Network, and other technologies can be leveraged to investigate the ecological functions and driving factors of microbial communities in forest ecosystems, as well as nutritional strategies, functional selections, and interspecific relations of saprophytic fungi.

## 8 Conclusions

Understanding how fine roots breakdown is important, toward explaining both how C and nutrients cycle, and ecosystem health and sustainable management practices as a whole. The quantities of C and N that are recycled through the development of fine roots and their subsequent decomposition is comparable to or even larger than, the aboveground plants component. Decomposition rate is correlated with the residence period of soil C and, consequently, fine root decomposition is a significant contributor to the global C budget. The production and death of fine roots, and the factors that affect them are important for energy flow and nutrient cycling in forest ecosystems. Yet they are still not well understood largely because the methods used to study them are limited. It is also difficult to summarize this complex process because the rate of fine root decomposition is impacted by a wide variety of variables. Climate (such as MAT, MAP, and altitude), substrate quality, microorganisms (such as saprophytic fungus), and soil features are all important in affecting the rates of litter decomposition. Researchers have yet to establishe a system that simultaneously accounts for all these variables. However, considering the increasing anthropogenic impacts on biogeochemical cycles, research into fine root decomposition is essential. This research examines the elements that contribute to fine root degradation, the degradation pattern of biomass materials, and the techniques used to investigate fine root degradation. Knowledge from this area of inquiry will help us plan for sustainable land use and forestry, and will contribute to our environments long-term productivity and wellness for coming generations.

## Author contributions

SS: Conceptualization, Writing – original draft, Writing – review & editing. LH: Writing – original draft. MK: Writing – original draft, Writing – review & editing. HW: Writing – original draft, Writing – review & editing. DH: Writing – original draft, Writing – review & editing. XM: Writing – original draft, Writing – review & editing. TP: Writing – original draft, Writing – review & editing. BL: Writing – original draft, Writing – review & editing. MZ: Writing – original draft, Writing – review & editing. QL: Writing – original draft, Writing – review & editing. NS: Writing – original draft. RW: Writing – original draft. MI: Writing – original draft. PZ: Conceptualization, Writing – original draft, Writing – review & editing. HS: Conceptualization, Supervision, Writing – original draft, Writing – review & editing.
